# The microbiome-gut-brain axis: a new perspective on the pathogenesis and intervention of frailty

**DOI:** 10.3389/fcimb.2026.1859069

**Published:** 2026-06-26

**Authors:** Qianqian Su, Huiyan Niu

**Affiliations:** Department of Geriatrics, Shengjing Hospital of China Medical University, Shenyang, Liaoning, China

**Keywords:** aging, frailty, gut microbiome, intervention strategies, microbiome-gut-brain axis

## Abstract

With the acceleration of global aging, frailty syndrome has become an important public health challenge. This article reviews the new perspective of the microbiome-gut-brain axis (MGBA) in the pathogenesis and intervention strategies of frailty, and systematically analyzes the bidirectional association between the gut microbiome and frailty, the potential biological mechanisms, and the therapeutic progress targeting the microbiome. Evidence shows that microbiome-gut-brain axis (MGBA) plays a pivotal role in frailty, and its regulation not only helps to reveal the multidimensional nature of frailty, but also provides important directions for the development of novel biomarkers and personalized interventions. Multi-dimensional targeting of MGBA may be an effective way to promote healthy aging.

## 1 Introduction

With the acceleration of global population aging, frailty syndrome has become a core challenge in the field of public health, which is characterized by decreased physiological reserve and increased vulnerability to stress, and is closely related to adverse outcomes such as mortality, functional decline, and increased use of medical resources (Jiang et al., 2025). Epidemiological data indicates that frailty affects millions of older adults worldwide. A review encompassing 62 countries revealed a prevalence rate of 12% based on the frailty phenotype (FP). while prevalence based on FI reaches 24%. Frailty is associated with a 3.5-fold increase in postoperative mortality, a 2.4-fold higher risk of non-home discharge, and a 50% rise in healthcare costs, highlighting its substantial economic burden (Faller et al., 2019; Alkadri et al., 2021). Recent studies further reveal that frailty prevalence significantly increases with age. For instance, among community-dwelling older adults, prevalence is 11% in those aged 50–59 years but rises to 51% in those aged 90 years and older. In high-risk settings such as acute care hospitals and nursing homes, prevalence approaches 50% (Kim and Rockwood, 2024). Although existing research has primarily focused on traditional risk factors such as malnutrition and chronic diseases, the mechanisms underlying frailty remain incompletely understood. There is an urgent need for in-depth exploration of its complexity from a multidimensional perspective.

Frailty is not a static state of a single dimension, but a dynamic and reversible multi-dimensional construction, involving the interaction of physiological, psychological, social and environmental fields (Jiang et al., 2025). At present, there is no unified definition of frailty. It is a complex syndrome characterized by the decline of multiple organ functions caused by sarcopenia, malnutrition, changes in hormone levels, and increased inflammation, leading to the weakening of the body’s ability to regulate stimulation and stress (Liu and Yang, 2024; Jiang et al., 2025; Johnson et al., 2025). In recent years, assessment tools have expanded from focusing on physical function (such as frailty phenotype) to covering multi-dimensional indicators such as cognition, nutrition and social support. 51 tools such as frailty index (FI) and clinical frailty Scale (CFS) have been developed, but there are still differences in their prediction accuracy and clinical applicability (Cesari et al., 2014; Faller et al., 2019; Oviedo‐Briones et al., 2022). This diversity of tools reflects the heterogeneity of frailty: the systematic review by Buta et al. (2016) identified 67 frailty assessment tools, among which Fried frailty phenotype (PFP) was the most commonly used in the research literature, but there were significant differences in conceptual basis and feasibility among the tools (Rozenberg et al., 2020). Electronic health data-based tools, such as the electronic Frailty Score (eFS), have shown good predictive agreement with clinical phenotypes, offering the potential for large-scale screening (Le Pogam et al., 2022). In specific populations such as elderly cancer survivors, tools such as FRAIL scale may need to modify cut-off points to optimize sensitivity and specificity, emphasizing that assessment needs to be context-integrated (Cheung et al., 2024). This multi-dimension reflects the heterogeneity of frailty, emphasizing the need to integrate biopsychological and social factors to reveal its underlying pathological mechanisms.

**Figure 1:**
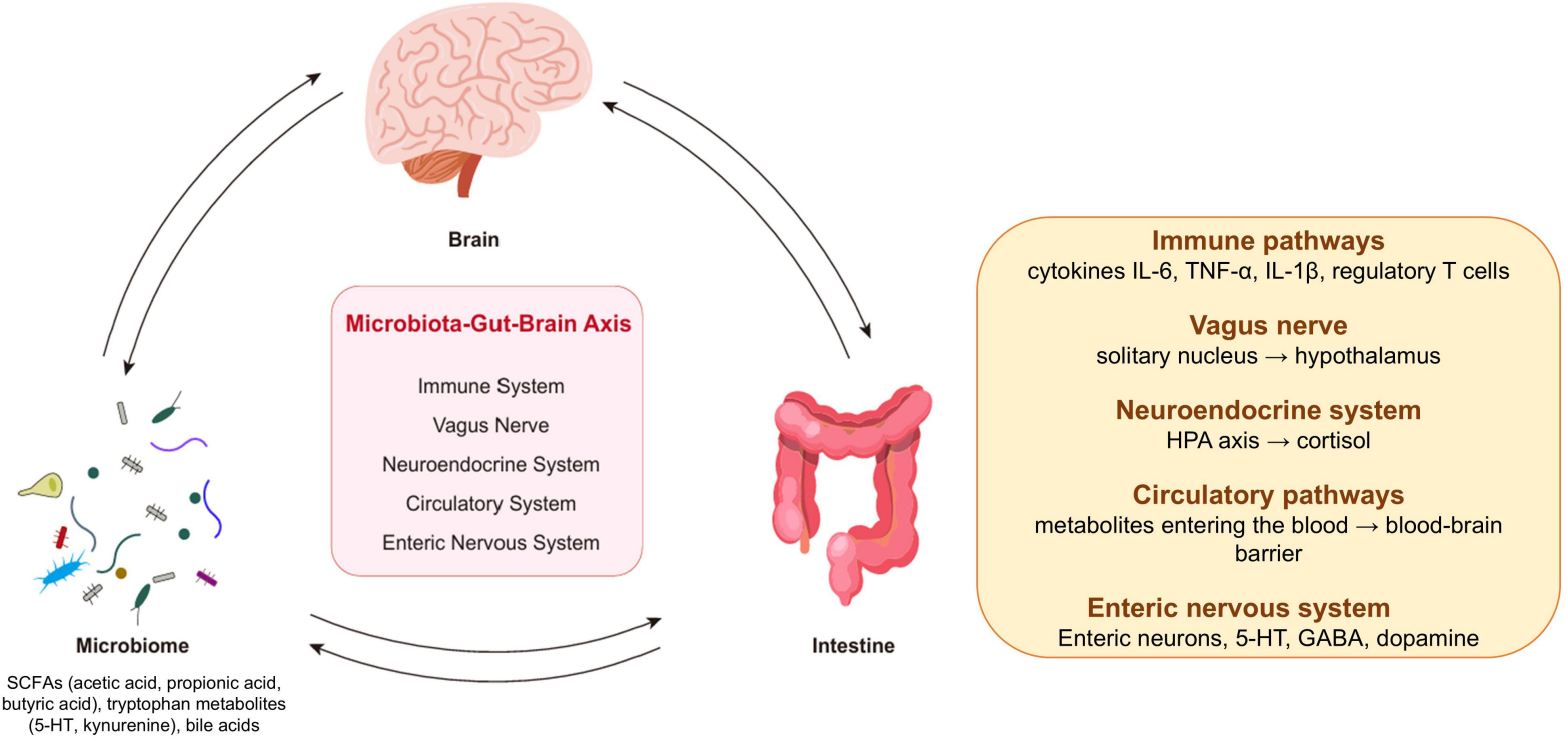
The bidirectional microbiota-gut-brain axis (MGBA) in frailty pathogenesis. Gut microbiota and metabolites regulate neuroinflammation, endocrine function, and metabolic homeostasis via neural, immune, and humoral pathways, thereby promoting age-related functional decline, frailty, and cognitive impairment. Aging and frailty further disrupt gut microbial homeostasis, forming a vicious cycle that aggravates systemic deterioration. Key components include immune pathways (cytokines IL-6, TNF-α, IL-1β, regulatory T cells), vagus nerve signaling (solitary nucleus → hypothalamus), neuroendocrine pathways (HPA axis → cortisol), circulatory pathways (metabolites entering the blood → blood-brain barrier), and the enteric nervous system (enteric neurons, 5-HT, GABA, dopamine). Aging and frailty further disrupt gut microbial homeostasis (reduced SCFAs, altered tryptophan metabolites, dysregulated bile acids), forming a vicious cycle that aggravates systemic deterioration. MGBA, microbiota-gut-brain axis; SCFAs, short-chain fatty acids; 5-HT, serotonin (5-hydroxytryptamine); IL-6, interleukin-6; TNF-α, tumor necrosis factor-α; IL-1β, interleukin-1β; Tregs, regulatory T cells; NTS, solitary nucleus (nucleus tractus solitarii); HPA, hypothalamic-pituitary-adrenal; BBB, blood-brain barrier; ENS, enteric nervous system; GABA, gamma-aminobutyric acid; DA, dopamine.

In recent years, the MGBA has emerged as a key framework revealed to play a pivotal role in the pathogenesis of frailty. The MGBA functions as a bidirectional homeostatic system connecting the gastrointestinal tract, central nervous system, and muscular systems, involving autonomic nerves, the hypothalamic-pituitary-adrenal axis, endocrine, and immune signaling pathways (**Figure 1**) (Di Sabatino et al., 2018; Cryan et al., 2019; Chakrabarti et al., 2022; Schneider et al., 2024). Frail individuals often exhibit deteriorated gut function, such as increased intestinal permeability, dysbiosis, and low-grade chronic inflammation (inflamm-aging). These alterations may exacerbate declining physiological reserves via the MGBA (Di Sabatino et al., 2018; Pan et al., 2025a). The reduced gut microbiota diversity associated with frailty correlates with increased opportunistic pathogens (e.g., Enterobacteriaceae), which further promote frailty progression through immune activation and neuroinflammatory pathways (Di Sabatino et al., 2018; Haran and McCormick, 2021). Dysregulation of MGBA signaling mechanisms—such as microbial metabolites modulating neurotransmitters and immune responses—in frailty-related diseases (e.g., Parkinson’s disease and Alzheimer’s disease) underscores its potential as a therapeutic target (Long-Smith et al., 2020). Preliminary evidence suggests that the microbiome-gut-brain axis plays a role in frailty, and its regulation may help reveal the multidimensional nature of this condition while offering potential directions for biomarker development and personalized interventions. However, current findings are primarily derived from cross-sectional studies and animal models, and further validation through large-scale longitudinal studies and randomized controlled trials is needed before clinical application can be recommended. Frailty exhibits a bidirectional vicious cycle with multidimensional risk factors such as inflammation, physical inactivity, and social isolation. Frailty-related factors—including comorbidity and functional decline—exacerbate physiological dysregulation, while early interventions like exercise and nutritional support can reverse the frailty process (Jiang et al., 2025). The biological mechanisms of frailty involve multisystem physiological dysregulation, including accelerated aging processes such as chronic inflammation, mitochondrial dysfunction, and cellular senescence. These mechanisms increase vulnerability by impairing physiological reserves (Kim and Rockwood, 2024). Intervention strategies like exercise, nutritional supplementation, and comprehensive geriatric assessments have demonstrated improvements in frailty status, but their implementation outcomes in routine care settings have been inconsistent, suggesting the need for personalized approaches (Kim and Rockwood, 2024).

From a geroscience perspective, aging is driven by a set of interconnected hallmarks, including chronic inflammation, mitochondrial dysfunction, epigenetic alterations, cellular senescence, and dysbiosis. These hallmarks do not operate in isolation but rather interact within a complex network that amplifies age-related physiological decline. Within this framework, gut dysbiosis serves as both a consequence and a driver of aging, intersecting with multiple other hallmarks to promote systemic deterioration. The MGBA can be viewed as a specialized communication network through which gut dysbiosis connects to brain aging, neuroinflammation, and cognitive decline (López-Otín et al., 2023). This perspective helps clarify that while certain MGBA-related mechanisms, such as systemic inflammation and mitochondrial dysfunction, represent broad aging hallmarks affecting multiple organ systems, other pathways including vagal signaling and the production of neuroactive metabolites constitute more axis-specific routes of gut-brain communication. Thus, the MGBA operates at the intersection of systemic aging biology and organ-specific signaling, and understanding this dual nature is essential for developing targeted interventions. The following sections will systematically examine the clinical evidence linking the gut microbiome to frailty, explore the biological mechanisms through which MGBA pathways drive frailty progression, and evaluate therapeutic strategies targeting this axis.

This review will systematically summarize clinical evidence, focusing on the critical role of the MGBA in the pathogenesis of frailty. It will examine its association with frailty effects through immune-inflammatory, mitochondrial metabolic, and neuroendocrine pathways, evaluate the efficacy of gut microbiota-targeted interventions (such as dietary modifications, probiotics, and fecal microbiota transplantation), and discuss future research directions. By emphasizing the dynamic regulation of the MGBA and the multidimensional nature of frailty, this paper aims to provide new perspectives for frailty prevention and management, thereby advancing healthy aging practices.

## 2 Frailty and gut microbiome: clinical evidence and bidirectional link

### 2.1 Association between frailty phenotype and gut microbiota composition

With the progress of multi-omics technology, the role of gut microbiome in the pathogenesis of frailty has attracted increasing attention as a key mediator between environmental factors and host physiological decline. Recent studies have systematically revealed the diversity and taxonomic composition of gut microbiota in frailty using metagenomics methods, which provides a new perspective for understanding microbiota-host interactions. Detailed data and results are provided in **Table 1**, which summarizes the sample characteristics, assessment tools, and key findings of the key studies. A review based on the evidence is presented below.

**Table 1:** Summary of key studies on the association between frailty and gut microbiome.

Author (year)	Study type	Sample characteristics	Cohort design	Frailty assessment tool	Alpha-diversity changes	Beta-diversity changes	Key microbial taxa changes	Ref.
Jiao et al. (2025)	Longitudinal mouse model	Animal model	Longitudinal study	modified Clinical Frailty Index (CFI)	ACE and Chao1 indices significantly decreased in the aging phase (p<0.001), rebounded in the frailty phase	Microbial communities significantly separated along PC1 (71.23% variance) in the frailty stage (PERMANOVA, p<0.001)	Enrichment of Lachnospiraceae_NK4A136_group in the frailty phase; significant decrease in butyrate and other SCFAs levels (p<0.01)	(Jiao et al., 2025)
Pu et al. (2025)	Human cohort analysis	n=1225, mean age 78.2 years	Cohort analysis	Frailty Index (FI)	Poor sleep quality not directly associated with alpha-diversity; FI associated with reduced microbial evenness	Beta-diversity at species and pathway levels significantly associated with sleep quality (PERMANOVA, p=0.01)	Faecalibacterium prausnitzii decreased in individuals with poor sleep quality, its abundance negatively correlated with FI (p<0.05)	(Pu et al., 2025)
Jarmukhanov et al. (2024)	Cross-sectional study	n=158 adults	Cross-sectional study	Frailty Index Score (FIS)	Simpson (p=0.004) and Shannon (p=0.011) indices significantly decreased; marginally significant after adjustment (padj=0.06)	Significant differences in community structure at phylum level (PERMANOVA, F = 2.2515, padj=0.019), not significant at species level	Decreased Firmicutes/Bacteroidetes ratio positively correlated with frailty (p<0.018); Oscillospiraceae enrichment associated with decreased mobility (padj=0.013)	(Jarmukhanov et al., 2024)
Almeida et al. (2022)	Systematic Review & Meta-analysis	11 studies (n=1239 participants)	Systematic Review/Meta-analysis	Tools from original studies	No significant difference between frail and non-frail groups (Richness Index SMD=-0.147, 95%CI=-0.394, 0.100, p=0.243; Diversity Index SMD=-0.033, 95%CI=-0.315, 0.250, p=0.820)	–	Qualitative analysis revealed differences in relative abundance of nearly 50 bacterial taxa	(Almeida et al., 2022)
Margiotta et al. (2020)	Observational study	n=64 patients with chronic kidney disease	Cross-sectional study	Fried’s Frailty Phenotype (FFP)	No significant difference in alpha-diversity between frail and non-frail groups	Beta-diversity analysis showed differences in community structure	–	(Margiotta et al., 2020)
Mirfakhraee et al. (2024)	Cross-sectional study	n=37 (14 frail, 23 non-frail) older adults	Cross-sectional study	Frailty syndrome in the elderly	–	–	Significantly increased Prevotella/Bacteroidetes ratio in frail individuals aged ≥70 years (p=0.03)	(Mirfakhraee et al., 2024)
Naito et al. (2024)	Cross-sectional analysis	n=786 Japanese community-dwelling older adults (≥65 years)	Cross-sectional study	modified frailty index	No significant differences in Chao1 and Shannon indices	Beta-diversity showed separation between groups (PCoA based on Bray-Curtis distance, p<0.05)	–	(Naito et al., 2024)
Zhang et al. (2025)	Cross-sectional study	n=672 Chinese older adults (50 sequenced)	Cross-sectional study	Fried’s Frailty Phenotype (FFP)	No significant differences in alpha-diversity indices (Chao1 P = 1.00; Shannon P = 0.57)	Significant differences in beta-diversity (weighted UniFrac and Bray-Curtis distances, PCoA p<0.05)	–	(Zhang et al., 2025)
Lim et al. (2021)	Cohort study	n=176 Korean community-dwelling older adults (mean age 74.7 yrs)	Cohort design	Frailty Index (FI)	FI negatively correlated with Shannon diversity (p<0.05, FDR<0.2)	Beta-diversity significantly associated with frailty degree (Adonis R²=0.0098, FDR<0.2)	FI positively correlated with Bacteroides fragilis and Clostridium hathewayi (FDR<0.2); negatively correlated with Prevotella copri and Coprococcus eutactus (FDR<0.2)	(Lim et al., 2021)
Jackson et al. (2016)	Observational study	n=728 female twins (age 42–86 years)	Large-scale observational study	Frailty Index (FI)	Frailty index negatively correlated with alpha-diversity (Shannon index p=9.54E-07)	–	Significant decrease in Faecalibacterium prausnitzii (p<0.05); increase in Eubacterium dolichum and Eggerthella lenta (p<0.05)	(Jackson et al., 2016)
Xu et al. (2021)	Cross-sectional study	n=94 (47 frail, 47 control) community-dwelling older adults (mean age 80.72 yrs)	Cross-sectional study	Fried’s Frailty Phenotype (FFP)	No significant difference in alpha-diversity metrics (Shannon P = 0.568; Faith’s phylogenetic distance P = 0.331)	Significant separation of microbial structure between frail and healthy groups (Bray-Curtis distance, R = 0.361, P = 0.001)	Decrease in Faecalibacterium, Prevotella, Roseburia (P<0.05); increase in Parabacteroides, Akkermansia, Klebsiella (P<0.05); Faecalibacterium negatively correlated with IL-6, Escherichia/Shigella positively correlated with IL-6	(Xu et al., 2021)
Wen et al. (2024)	Systematic Review	11 observational studies (total n=912 older adults)	Systematic Review	Tools from original studies	Some studies reported decreased alpha-diversity in frail individuals, but results were inconsistent (only 2 studies showed significant decrease)	Some studies reported increased beta-diversity, but results were inconsistent (only 2 studies showed significant increase)	Genera Prevotella, Faecalibacterium, Roseburia generally decreased; Bifidobacterium, Oscillospira etc. increased; Phyla Actinobacteria and Proteobacteria enriched, Firmicutes depleted	(Wen et al., 2024)
Zhang et al. (2020)	Cross-sectional study	n=27 (15 frail, 12 control) hospitalized older patients (mean age 81.63 ± 7.90 yrs)	Cross-sectional study	Frailty Index (FI), FI≥0.25 defined frail	No significant difference in alpha-diversity (Shannon p=0.722; Simpson p=0.887)	Beta-diversity showed a wider distribution range for the frail group in PCoA and NMDS plots (greater community structure heterogeneity)	4 families and 17 genera significantly different (p<0.05), e.g., Eubacterium decreased (p=0.023), Prevotella increased	(Zhang et al., 2020)
Vega-Abellaneda et al. (2025)	Observational study	Patients with cirrhosis	–	Liver Frailty Index (LFI)	–	–	Rothia dentocariosa positively correlated with LFI (r=0.57, p<0.001)	(Vega‐Abellaneda et al., 2025)

Regarding α diversity, research findings exhibit significant inconsistency, suggesting that changes in microbial richness and evenness may not be specific markers of frailty. Multiple studies report associations between frailty and reduced α diversity. For instance, Jiao et al. (2025) demonstrated biphasic changes in α diversity during aging using a longitudinal mouse model, while Pu et al. (2025) found that the FI correlates with decreased microbial evenness in a human cohort. Jarmukhanov et al. (2024) also supported this association through a cross-sectional study showing significant reductions in Simpson and Shannon indices with increasing frailty severity. However, this association is not universal: A systematic review and meta-analysis by Almeida et al. (2022) (encompassing 11 studies) quantitatively synthesized that no significant differences in α diversity indices were observed between frail and non-frail groups. Similarly, Margiotta et al. (2020) in patients with chronic kidney disease, Naito et al. (2024) in community-dwelling elderly Japanese individuals, and Zhang et al. (2025) and Xu et al. (2021) in their respective cohorts did not observe significant alterations in α diversity. This inconsistency may stem from study design heterogeneity, sample size limitations (e.g., the small sample size in Mirfakhraee et al. (2024)), or confounding factors (e.g., age, comorbidities, and medication use). As noted in the systematic review by Wen et al. (2024), only a minority of studies demonstrated a significant reduction in alpha diversity. Therefore, reduced alpha diversity is more likely to reflect aging or disease-related changes rather than being specific to frailty, suggesting that contextual factors must be carefully considered when interpreting such metrics.

In contrast, β-diversity analyses more consistently revealed a restructuring of microbial community architecture associated with frailty. Multiple studies using principal coordinate analysis (PCoA) or similar methods confirmed significant separation between the gut microbial composition of frail individuals and non-frail groups. For instance, Jiao et al. (2025) observed distinct separation along the PC1 axis in mouse models during the frailty phase; Pu et al. (2025) reported species- and pathway-level β-diversity correlations with sleep quality; Jarmukhanov et al. (2024) identified phylum-level structural differences; and Naito et al. (2024), Zhang et al. (2025)), Lim et al. (2021), and Xu et al. (2021) all demonstrated significant associations between β-diversity and frailty status in human cohorts. This consistency suggests that alterations in microbial community structure (rather than mere abundance changes) may be more closely linked to frailty, reflecting the holistic characteristics of ecological imbalance. However, the extent of β-diversity changes varies across studies, indicating that its sensitivity may be influenced by taxonomic levels or distance metrics. Nevertheless, the overall evidence supports the association between frailty and microbial ecological imbalance.

Changes in key microbial communities further elucidate the physiological significance of frailty-associated dysbiosis. Studies consistently report reductions in beneficial bacteria and increases in pro-inflammatory bacteria, forming a “dysbiosis index” pattern. Short-chain fatty acid (SCFA)-producing symbionts such as Faecalibacterium prausnitzii are frequently depleted in frail individuals (Jackson et al., 2016; Pu et al., 2025), with their abundance negatively correlated to inflammatory markers like IL-6 (Xu et al., 2021). SCFA producers including Roseburia and Prevotella also show declining trends (Xu et al., 2021; Wen et al., 2024). Conversely, pro-inflammatory microbiota like Bacteroidetes, Eubacterium dolichum, and Eggerthella lenta increase (Jackson et al., 2016), and opportunistic pathogens such as Bacteroides fragilis and Clostridium hatchewayi positively correlate with frailty indices (Lim et al., 2021). Zhang et al. (2020) found that despite similar dominant microbial compositions, detailed analysis revealed significant differences in multiple bacterial families and genera. For instance, SCFA-producing Eubacterium decreased while pro-inflammatory Prevotella increased, further supporting the notion that impaired SCFA metabolism may exacerbate inflammation and frailty progression. However, Faecalibacterium prausnitzii showed no differences, suggesting that microbial changes are not uniform. Additionally, phylum-level alterations revealed enrichment of Actinobacteria and Proteobacteria alongside depletion of Firmicutes (Wen et al., 2024). Disease-specific features, such as Rothia dentocariosa positively correlating with the hepatic frailty index in cirrhosis patients (Vega‐Abellaneda et al., 2025), underscore the context-dependent nature of microbial shifts. Contradictions persist, however: for instance, Akkermansia increased in frail groups in Xu et al.’s (Xu et al., 2021) study, despite its conventionally beneficial status, suggesting potential influences from population, diet, or methodology on microbial function. Collectively, these alterations point toward impaired SCFA metabolism and weakened intestinal barrier function, potentially accelerating frailty progression by exacerbating systemic inflammation (D’Amico et al., 2023). However, establishing causality requires validation through longitudinal studies.

In summary, the association between frailty and gut microbiota presents a complex picture: α diversity changes are inconsistent, potentially influenced by multidimensional confounding factors; β diversity alterations are more consistent, indicating community structural reorganization; and dysbiosis of key microbial groups is characterized by a reduction in beneficial bacteria and an increase in pro-inflammatory bacteria (Margiotta et al., 2020; Wang and Wu, 2021; Strasser and Ticinesi, 2023). These findings underscore the potential of the microbiome as a biomarker for frailty. However, existing evidence primarily relies on cross-sectional designs, making causal inference challenging. Future research should integrate longitudinal multi-omics data, control for confounding factors, and conduct intervention studies to elucidate mechanisms. This will lay the groundwork for developing targeted intervention strategies that leverage the MGBA.

### 2.2 Bidirectional interaction between frailty and the gut microbiome: from causal mechanisms to biomarker applications

Although cross-sectional studies reveal strong correlations between frailty and gut dysbiosis, these findings suggest association rather than causation (Casati et al., 2019). In recent years, causal inference methods such as Mendelian randomization (MR) have gradually shifted evidence from correlation toward bidirectional causality, revealing a potential self-reinforcing cycle between frailty and the microbiome. This section will systematically evaluate the mechanistic evidence for bidirectional interactions and, based on this foundation, explore the potential of the microbiome as a biomarker for frailty, emphasizing the translational logic from causal understanding to clinical application.

In the causal direction of frailty-driven dysbiosis, MR studies provide genetic-level support. For instance, Bo et al. (2024) revealed through reverse MR analysis that genetic predisposition to FI significantly increased Butyrivibrio abundance, suggesting frailty may directly alter gut microbial composition. Similarly, multiple studies indicate that frailty frequently co-occurs with environmental factors such as sedentary behavior, polypharmacy, and malnutrition. These factors can independently induce dysbiosis, such as reducing microbial diversity and promoting pathogen overgrowth (Ticinesi et al., 2019a; O’Toole et al., 2023). Collectively, these findings suggest that frailty and its associated phenotypes may preferentially affect specific microbial communities by altering host metabolic and immune states, rather than merely representing consequential changes.

In contrast, evidence for the mechanism by which dysbiosis exacerbates frailty via the MGBA is more direct. Metric regression analysis revealed that increased gene prediction of specific microbial groups such as Betaproteobacteria and Allisonella correlates with frailty risk, while Bacteroidetes exerts a protective effect. Inverse variance-weighted models confirmed the robustness of this causal association (Hou et al., 2025; Yan et al., 2025). Mechanistically, disrupted gut metabolites like short-chain fatty acids (SCFAs) can influence neuroendocrine, inflammatory, and muscle function through MGBA pathways (Pan et al., 2025b); The phylum Bacteroidetes and Eubacterium ruminantium exert protective effects against frailty, whereas genera like Betaproteobacteria and Bifidobacterium increase frailty risk (Bo et al., 2024; Wang et al., 2024). Furthermore, genetic association studies further support the link between genera such as Christensenellaceae R-7 and frailty, suggesting they may influence host health through pathways like SCFA production (Cui et al., 2023). These findings, validated through sensitivity analyses that ruled out pleiotropy and confounding, highlight dysbiosis as an independent etiological factor driving frailty progression through multisystem interactions.

Integrating bidirectional evidence suggests a potentially self-reinforcing cycle between frailty and dysbiosis. Magnetic resonance imaging (MRI) findings support bidirectional causality, indicating that frailty is influenced by microbiota while simultaneously affecting microbial composition, forming a feedback loop (Bo et al., 2024). Environmental factors like diet and exercise can simultaneously modulate frailty and microbiota, but dysbiosis may amplify frailty’s physiological deficits. For instance, reduced SCFA production exacerbates insulin resistance and muscle wasting, while frailty-related activity decline further deteriorates microbial diversity (Ticinesi et al., 2019a). This bidirectional interaction not only highlights the central role of MGBA in frailty but also provides a target for intervention strategies—namely, breaking this vicious cycle through microbiota regulation.

Based on the aforementioned causal mechanisms, microbiome characteristics demonstrate significant potential as biomarkers of frailty. Existing evidence indicates that microbiome features are not only associated with frailty status but may also hold predictive value for disease progression (Picca et al., 2020; Pu et al., 2024; Lu et al., 2025). For instance, plasma TMAO levels—as a gut microbiota-dependent metabolite—exhibit an independent association with frailty and a linear dose-response relationship, suggesting its potential as an effective predictive indicator (He et al., 2020). Similarly, specific microbial and metabolic markers (e.g., aspartate and threonine) identified through machine learning models such as the SO-CovSel method can distinguish frail from non-frail individuals with high accuracy, achieving classification rates of 91.7% and 87.5%, respectively (Picca et al., 2019). Collectively, these findings demonstrate that advanced models like ensemble learning can leverage microbiome data to construct robust predictive tools whose performance rivals traditional clinical indicators. Furthermore, the development of the gut microbial aging clock (gAge) further confirms that age prediction based on multi-view microbiome features is significantly correlated with frailty indices. In prospective cohorts, baseline microbial characteristics were shown to independently predict future frailty risk, highlighting their predictive value (Wang et al., 2024).

However, the clinical translation of microbiome biomarkers remains challenging. Heterogeneity exists in the frailty-associated microbial features reported across studies, such as inconsistent changes in the abundance of certain bacterial genera across populations, potentially stemming from sample size, population differences, or methodological issues (Margiotta et al., 2020). Despite prospective evidence, frailty develops slowly, making residual confounding factors difficult to fully exclude (Verdi et al., 2018). In clinical practice, high costs, lack of standardized protocols, and complex data interpretation limit the widespread adoption of microbiome analysis (Bautista et al., 2025). In contrast, traditional inflammatory markers like IL-6 and CRP are more readily detectable, but the microbiome may offer earlier warning signs or reveal distinct pathophysiological mechanisms, such as the gut-muscle axis pathway. Overall, microbiome biomarkers demonstrate significant potential in frailty management, yet their application in personalized medicine requires addressing heterogeneity and translational challenges.

In summary, bidirectional interaction mechanisms provide the theoretical foundation for the microbiome as a frailty biomarker, while advances in machine learning models accelerate its clinical translation. Future research should integrate longitudinal multi-omics data, clarify causal temporal relationships, and conduct intervention studies to validate MGBA-targeting strategies.

## 3 Potential biological mechanisms linking frailty and the microbial-gut-brain axis headings

The MGBA is defined as a bidirectional, multi-channel communication system that links the gut microbiota to the central nervous system through neural, endocrine, immune, and metabolic pathways. It is critical to distinguish MGBA-specific mechanisms from the generalized systemic effects of gut dysbiosis. Systemic effects, such as increased intestinal permeability, bacterial translocation, and chronic low-grade inflammation, represent non-specific consequences of microbial imbalance that can affect multiple organs including the brain. In contrast, MGBA-specific mechanisms involve direct signaling pathways that enable gut-brain crosstalk independent of systemic circulation. These include vagal afferent transmission, hypothalamic-pituitary-adrenal axis modulation, and microbially-derived neuroactive metabolites that cross the blood-brain barrier. In the following sections, we first delineate the systemic inflammatory consequences of dysbiosis that contribute to frailty, then focus on MGBA-specific pathways that provide more direct mechanistic links between gut ecology and brain function.

### 3.1 Immune and inflammatory pathways

Existing research indicates that inflammation serves as a key bridge connecting the MGBA to frailty. At its core lies “inflammatory aging,” an age-related chronic state of low-grade systemic inflammation characterized by persistently mild elevations in circulating pro-inflammatory cytokines such as IL-6 and CRP, which are closely associated with the onset and progression of frailty (Soysal et al., 2016; Ferrucci and Fabbri, 2018; Perazza et al., 2022; Taylor et al., 2023; Pan and Ma, 2024). In recent years, evidence has increasingly pointed to the gut microbiome and its associated intestinal barrier dysfunction as significant sources of chronic inflammation, sparking in-depth exploration of how the microbiota drives frailty through immune pathways (Li et al., 2023). Collectively, these findings indicate that inflammaging plays a central role in frailty, with gut microbial dysbiosis potentially serving as its initiating factor.

At the starting point of mechanism, dysbiosis of flora first leads to the impairment of intestinal barrier function, that is, “intestinal leakage” phenomenon. Healthy gut microbiota, such as butyrate-producing bacteria, may strengthen gut barrier function by regulating tight junction proteins and stimulating mucus secretion. However, dysbiosis of flora can destroy the mucus layer and tight junctions, increase intestinal permeability, and lead to “intestinal leakage” (Yuan et al., 2021). However, dysbiosis can disrupt this balance, and as described by Ferrucci et al (Ferrucci and Fabbri, 2018), age-related changes in microbial composition such as a decrease in beneficial bacteria and an increase in pathogenic bacteria can cause thinning of the mucus layer and downregulation of tight junction protein expression, thereby increasing intestinal permeability. However, dysbiosis of the flora, such as decreased Firmicutes and overgrowth of Proteobacteria, is associated with increased intestinal permeability (as indicated by elevated serum zonulin), possibly through as yet undefined mechanisms of barrier damage (Rashidah et al., 2022).

The core link involves bacterial translocation and activation of systemic inflammation. When the intestinal barrier is damaged, bacterial products such as lipopolysaccharide (LPS) are translocated into the circulation and promote the release of pro-inflammatory factors such as IL-1β and IL-6 by activating Toll-like receptors and NLRP3 inflammasome, thus forming a chronic inflammatory state (Ferrucci and Fabbri, 2018; Yuan et al., 2021). In the frail population, a cross-sectional study by Bålsrud et al. (2024) showed that serum levels of inflammatory markers, such as IL-6, were positively correlated with the frailty index. Similarly, animal studies have shown that microbiota depletion decreases circulating inflammatory monocytes with improvement in frailty symptoms (Conn et al., 2025). Serum high mobility group protein B1 (HMGB1) levels are significantly increased in frail people, and are associated with intestinal barrier damage and systemic inflammation. These changes are consistent with the severity of frailty syndrome (Rashidah et al., 2022). These lines of evidence collectively suggest that bacterial translocation serves as a central bridge linking intestinal dysbiosis to systemic inflammation.

Inflammatory effects not only directly impact core manifestations of frailty—such as accelerating muscle decline by promoting protein catabolism, inhibiting anabolism, and impairing mitochondrial function—but also influence the central nervous system by inducing neuroinflammation, leading to feelings of fatigue and cognitive decline (Ferrucci and Fabbri, 2018; Rashidah et al., 2022; Wang et al., 2024; Azzolino et al., 2025). Oxidative stress and inflammation synergistically accelerate muscle atrophy and the progression of sarcopenia in frailty (Dzięgielewska-Gęsiak and Muc-Wierzgoń, 2023). Concurrently, inflammation affects the central nervous system; Pan and Ma (2024) noted that IL-6 and TNF-α can cross the blood-brain barrier to induce neuroinflammation, disrupting neurotransmitter balance and thereby contributing to frailty symptoms like fatigue, cognitive decline, and depression. Inflammation induces mitochondrial dysfunction and oxidative stress, impairing energy metabolism and exacerbating physical decline (Ticinesi et al., 2017). Collectively, these mechanisms demonstrate that inflammation directly drives the pathophysiology of frailty through multiple pathways.

In summary, inflammatory aging, as a chronic low-grade systemic inflammatory state, plays a central role in the onset and progression of frailty. Its origins are closely linked to dysbiosis of the gut microbiome and the resulting impairment of barrier function. Microbiome imbalance leads to translocation of bacterial byproducts, activating systemic inflammatory pathways. This, in turn, directly exacerbates frailty manifestations such as muscle wasting and cognitive decline by promoting protein degradation, suppressing anabolic processes, and inducing neuroinflammation. Collectively, these findings elucidate inflammation’s pivotal role in driving the multisystem pathophysiology of frailty, underscoring the critical significance of immune and inflammatory pathways in age-related health decline. Conflicting findings across microbiome-frailty studies may be explained by differences in cohort characteristics, including chronological age distribution, underlying biological aging trajectories, physical activity levels, diet, medication use, particularly antibiotics and proton pump inhibitors, social isolation, cognitive status, metabolic comorbidities, and polypharmacy. Each of these variables independently shapes both gut microbiota composition and frailty expression. For example, the association between microbial diversity and frailty indices is more pronounced in females, suggesting sex-specific biology. Stratifying by these contextual factors in future studies may help reconcile apparently contradictory results and identify distinct microbiome-frailty subtypes.

It is noteworthy that systemic low-grade inflammation, also known as inflammaging, which is triggered by gut dysbiosis, forms the foundation of the pathophysiology of frailty. However, from the perspective of the microbiota-gut-brain axis, this systemic inflammation is not merely an independent negative event. Circulating inflammatory cytokines such as interleukin-6 and tumor necrosis factor-alpha can transmit peripheral inflammatory signals to the central nervous system either through active transport across the blood-brain barrier or by acting on the vascular endothelial cells of the blood-brain barrier. Therefore, the immune pathway plays a dual role in this context. It serves not only as a non-specific systemic effector through which gut dysbiosis leads to multi-organ dysfunction, including muscle impairment, but also as a key messenger that activates the microbiota-gut-brain axis, induces neuroinflammation, and contributes to central symptoms of frailty such as fatigue and cognitive decline.

### 3.2 Melatonin and tryptophan metabolism: the molecular bridge connecting gut microbiota, circadian rhythm and decline

Melatonin is an indoleamine hormone synthesized by the pineal gland that regulates circadian rhythms by activating MT1 and MT2 receptors in the suprachiasmatic nucleus of the hypothalamus. A systematic review encompassing 19 studies systematically evaluated the physiological levels of melatonin in healthy older adults. The results showed that the mean nighttime peak melatonin concentration was 49.3 pg/mL in participants with a mean age of 65 to 70 years, whereas this concentration decreased to 27.8 pg/mL in participants aged 75 years and older, a difference that was statistically significant (Scholtens et al., 2016). The study further noted that although 24-hour total melatonin production did not change significantly during healthy aging, the nighttime peak concentration exhibited a marked declining trend. A bidirectional regulatory relationship exists between melatonin and the gut microbiota. An animal study using 16S rRNA sequencing and metabolomic analysis confirmed that oral melatonin administration dose-dependently inhibited the ileal FXR signaling pathway and the downstream transcriptional levels of Shp and Fgf15 in aged mice, significantly reducing triglyceride and cholesterol absorption and increasing serum and fecal melatonin levels (Liu et al., 2024). Mechanistically, melatonin modulates gut microbiota-mediated bile acid metabolism, thereby reducing hepatic trimethylamine N-oxide production and lipid metabolism abnormalities. Depletion of the gut microbiota with antibiotics abolished the inhibitory effect of melatonin on the FXR signaling pathway, indicating that melatonin regulation of the FXR pathway is dependent on the gut microbiota. These findings provide molecular targets for frailty intervention, as exogenous melatonin supplementation may compensate for age-related secretory deficiencies. Melatonin was included in the Chinese Health Food Ingredient Directory in 2021, with a recommended daily dosage of 1 to 3 mg, providing a safety basis for its use in frailty interventions.

Tryptophan is an essential amino acid for humans, and its metabolism proceeds through three primary pathways: the kynurenine pathway, the serotonin pathway, and the direct conversion pathway. A study using human cell lines, mouse models, and Drosophila models revealed for the first time the central role of SIRT6 in maintaining tryptophan metabolic balance. As an NAD+-dependent histone deacetylase, SIRT6 determines the direction of tryptophan metabolism by regulating the transcription of key tryptophan-metabolizing genes such as TDO2 and AANAT. In SIRT6-knockout cells, the kynurenine-to-tryptophan ratio increased, the serotonin-to-tryptophan ratio decreased, and NAD+ levels were significantly reduced (Kaluski-Kopatch et al., 2025). Aging leads to decreased SIRT6 activity, shifting tryptophan metabolism toward the kynurenine pathway and resulting in accumulation of the neurotoxic metabolite quinolinic acid. In a SIRT6-deficient Drosophila model, TDO2 inhibition successfully reversed the accumulation of neurotoxic metabolites, protected brain tissue, and improved neuromotor behavior. Another review systematically summarized the role of kynurenine pathway metabolites in the pathogenesis of frailty, noting that chronic low-grade inflammation, or inflammaging, activates indoleamine 2,3-dioxygenase, which increases the degradation of tryptophan toward the kynurenine pathway. The accumulation of active metabolites from this pathway sustains and exacerbates the aging process through their immunomodulatory, pro-inflammatory, and cytotoxic properties. A QPRT-knockout mouse model further demonstrated that dysregulation of the kynurenine pathway leads to reduced locomotor activity, decreased lean body mass, lower oxygen consumption, and impaired glucose clearance, with these effects exhibiting sex-specific characteristics (Sultana et al., 2023). Approximately 1% to 2% of tryptophan is converted to serotonin by tryptophan hydroxylase, and about 90% of serotonin is synthesized by enterochromaffin cells in the gut, a process that depends on signaling molecules provided by the gut microbiota. Serotonin is subsequently converted to melatonin in the pineal gland via the enzymes aralkylamine N-acetyltransferase and hydroxyindole O-methyltransferase, forming the tryptophan-serotonin-melatonin metabolic axis (Gupta et al., 2023). These metabolic pathways constitute a self-reinforcing feedback loop: aging reduces SIRT6 activity, diverting tryptophan metabolism toward the kynurenine pathway, which in turn reduces serotonin and melatonin synthesis, exacerbates circadian rhythm disruption and sleep disturbances, and ultimately promotes frailty progression. A 12-week single-blind randomized controlled trial enrolled 30 older adults aged 66 ± 3 years to evaluate the effects of 5-hydroxytryptophan supplementation on sleep quality and gut microbiota. The results showed that among participants with poor sleep quality, the 5-hydroxytryptophan supplementation group experienced a 2.80-point improvement in subjective sleep scores, a significant increase in the Simpson diversity index of the gut microbiota, and an increased relative abundance of short-chain fatty acid-producing bacteria (Sutanto et al., 2024). These findings provide molecular targets for frailty intervention: tryptophan or 5-hydroxytryptophan supplementation can increase the supply of serotonin and melatonin precursors, and modulation of the gut microbiota can improve tryptophan metabolic balance.

The melatonin and tryptophan metabolic pathways discussed above exemplify the intricate interplay between systemic aging biology and MGBA specific communication. On one hand, the age-related decline in SIRT6 activity and the subsequent shift of tryptophan metabolism toward the kynurenine pathway represent systemic metabolic alterations that broadly affect multiple organs including muscle, liver, and brain, contributing to frailty through generalized inflammation and mitochondrial dysfunction. On the other hand, the gut microbiota-dependent production of serotonin and its conversion to melatonin, as well as the direct effects of tryptophan metabolites on vagal signaling and hypothalamic regulation, constitute more specific MGBA mechanisms. Notably, these two layers are not mutually exclusive but rather operate in parallel and reinforce each other. The systemic inflammatory state can activate indoleamine 2,3-dioxygenase, further diverting tryptophan away from serotonin production, while the resulting serotonin deficiency directly impacts central nervous system function through MGBA pathways. Therefore, the tryptophan-serotonin-melatonin axis serves as an ideal model for understanding how systemic aging processes and MGBA specific signaling converge to drive frailty progression. Future interventions targeting this axis, whether through tryptophan or 5-hydroxytryptophan supplementation, should be evaluated for their effects on both systemic metabolic outcomes and MGBA mediated neurological functions.

### 3.3 Mitochondrial dysfunction and oxidative stress

Beyond ATP production and ROS generation, mitochondria serve as central hubs for multiple cellular processes relevant to frailty. The tricarboxylic acid cycle provides intermediates for biosynthesis and supports anaplerotic reactions that maintain metabolic homeostasis. Mitochondria also regulate cellular proteostasis through the mitochondrial unfolded protein response and coordinate mitophagy to remove damaged organelles. Furthermore, mitochondrial-derived metabolites influence epigenetic modifications and post-translational regulation, connecting organellar function to nuclear gene expression. In the context of frailty, impairment of these non-canonical mitochondrial functions may contribute to sarcopenia through disrupted anabolic signaling, impaired protein quality control, and altered metabolic flexibility in skeletal muscle and neural tissues.

Building upon immune and inflammatory pathways, chronic inflammation itself has been demonstrated to exacerbate oxidative stress, thereby providing fertile ground for the development of frailty (El Assar et al., 2020). This section focuses on how the gut microbiota further contributes to frailty pathogenesis by directly regulating mitochondria—the core organelles governing energy metabolism. Existing evidence indicates that the gut microbiota serves as a key regulator of mitochondrial function, with SCFAs playing a central role as critical microbial metabolites (Ticinesi et al., 2019b). For instance, SCFAs (such as butyrate) can be produced by microbial fermentation of undigested carbohydrates and absorbed by colonic cells and other tissues. They indirectly support mitochondrial function by influencing energy metabolism and redox balance (Chen et al., 2022; Prajapati et al., 2025); Similarly, butyrate can enhance fatty acid oxidation and glucose metabolism by activating pathways such as FFAR2/3, AMPK/CPT-1, and PPARα, thereby indirectly supporting mitochondrial energy homeostasis (Chen et al., 2022; Chaudhary et al., 2025; Zhu et al., 2025). Ticinesi et al. (2019b) noted that gut microbiota produce SCFAs like butyrate, which can be absorbed and systemically regulate host metabolism—for instance, by improving insulin sensitivity and reducing inflammation—thereby indirectly contributing to energy homeostasis. Concurrently, butyrate treatment directly modulates mitochondrial membrane potential and ATP levels while reducing ROS accumulation, thereby alleviating cellular senescence (Souders et al., 2023; Rees et al., 2025). Collectively, these findings demonstrate that microbiota-derived metabolites play a crucial role in sustaining mitochondrial homeostasis.

However, dysbiosis disrupts this equilibrium, leading to a vicious cycle of mitochondrial dysfunction and oxidative stress. Reduced SCFAs trigger diminished mitochondrial respiratory efficiency and insufficient ATP production, subsequently inducing muscle protein breakdown and atrophy—directly linked to core frailty symptoms such as fatigue and sarcopenia (El Assar et al., 2020; Souders et al., 2023; Zhu et al., 2025). Inefficient mitochondria are more prone to producing and leaking excessive reactive oxygen species (ROS). When ROS exceed antioxidant defense capacities, oxidative stress is triggered, damaging mitochondrial DNA and proteins, further deteriorating mitochondrial function (Arauna et al., 2020; Chen et al., 2022; Wang et al., 2022; Rees et al., 2025); This process not only forms a positive feedback loop but also activates the NLRP3 inflammasome, cross-talking with inflammatory pathways to jointly drive inflammaging (Chen et al., 2022; Chaudhary et al., 2025). For instance, Arauna et al. (2020) observed elevated GDF-15 levels in frail elderly individuals, reflecting the cumulative effects of mitochondrial dysfunction and oxidative stress. The FRAMITO study further underscored mitochondrial DNA damage as a potential biomarker for frailty (Locatelli et al., 2025).

Linking the aforementioned mechanisms directly to the frailty phenotype highlights the central role of the microbiota-mitochondria axis: mitochondrial dysfunction synergizes with oxidative stress and inflammation to accelerate muscle protein degradation through pathways such as upregulation of Atrogin-1/MuRF1, explaining muscle atrophy and reduced endurance in sarcopenia (El Assar et al., 2020; Chen et al., 2022; Zhu et al., 2025). As the physical manifestation of frailty, sarcopenia is closely associated with mitochondrial functional decline. Alterations in microbiota metabolites (e.g., TMAO) exacerbate tissue damage through oxidative stress, thereby promoting frailty syndrome (Chen et al., 2022).

Given this finding, future research should delve deeper into how microbiota interventions reverse mitochondrial damage. However, current evidence primarily stems from cross-sectional studies, such as the FRAMITO protocol, which aims to validate the association between mitochondrial biomarkers and frailty through functional analysis (Locatelli et al., 2025). Longitudinal data confirming causal relationships remains lacking. Overall, the gut microbiota offers a novel perspective on understanding frailty by regulating mitochondrial function and oxidative stress. However, substantial experimental evidence is urgently needed to identify therapeutic targets.

Mitochondrial dysfunction and oxidative stress are core cellular mechanisms driving frailty. Although these processes are particularly prominent in peripheral tissues such as skeletal muscle, where they directly lead to sarcopenia and physical decline, their association with the MGBA can be understood at two levels. First, as a systemic consequence, systemic oxidative stress and mitochondrial damage exacerbated by gut dysbiosis can indirectly impair high-energy-demand brain function through mechanisms such as depleting systemic NAD+ levels and exacerbating inflammation. Second, as a synergistic amplification loop, gut microbial metabolites such as short-chain fatty acids can cross the blood-brain barrier and directly influence mitochondrial biogenesis in central neurons, thereby directly linking peripheral gut microbiota status to central energy metabolism. Therefore, mitochondrial dysfunction serves not only as a systemic aging pathway that operates independently of the microbiota-gut-brain axis but also as a potential downstream target through which this axis regulates neurological function and frailty status.

### 3.4 Endocrine and neuro-metabolic pathways

Recent studies have progressively revealed that the gut microbiota plays a pivotal role in the pathogenesis of frailty through endocrine and neuro-metabolic pathways. Beyond immune-inflammatory pathways, metabolic communication within the MGBA represents another critical link connecting dysbiosis to the pathophysiology of frailty (Ticinesi et al., 2023). Existing studies indicate that gut microbes directly influence host endocrine and neurometabolic balance by modulating the hypothalamic-pituitary-adrenal (HPA) axis, producing neuroactive substances, and secreting metabolites such as SCFAs (Dicks, 2022).

Regarding HPA axis regulation, studies in germ-free animals demonstrate that mice lacking gut microbiota exhibit impaired development and function of the nervous system, including stress-related responses. Re-colonization with gut microbiota or their metabolites (such as short-chain fatty acids) can help restore these functions (Dicks, 2022). Similarly, the production and metabolism of neuroactive substances are also regulated by the microbiota. For example, the majority of serotonin (5-HT) in the human body is produced by chromaffin cells in the gut, a process highly dependent on signals provided by the gut microbiota, such as SCFAs (Mhanna et al., 2024). 5-HT not only influences mood and cognition but also participates in gastrointestinal motility and sleep regulation, all of which are associated with frailty. Gut microbiota, including Lactobacillus, Bifidobacterium, and Bacteroides—which are more abundant in the human gut—can produce gamma-aminobutyric acid (GABA). GABA primarily acts to inhibit neural excitation and may influence anxiety levels (Braga et al., 2024; Mhanna et al., 2024). Tryptophan metabolism can generate neuroactive metabolites such as quinolinic acid (QUIN) and kynurenic acid (KYNA) through the kynurenine pathway, which are closely associated with inflammation and cognitive function (Al Saedi et al., 2022). However, whether neuroactive substances produced by gut microbiota (e.g., GABA) can directly cross the blood-brain barrier to influence the central nervous system remains controversial. Their effects may be mediated more indirectly through pathways such as vagal afferents, immune regulation, or influencing the homeostasis of central neuroactive substances (e.g., GABA/glutamate ratio) (Braga et al., 2024; Mhanna et al., 2024).

In terms of direct regulation of microbial metabolites, SCFAs serve as a vital energy source for intestinal epithelial cells and enter the peripheral circulation to fuel tissues such as muscle (Kasubuchi et al., 2015). As inhibitors of histone deacetylases (HDACs) and ligands for G protein-coupled receptors (e.g., FFAR2, FFAR3), SCFAs regulate multiple physiological processes, including gut hormone secretion (e.g., GLP-1, PYY), lipid metabolism, and inflammatory responses (Kasubuchi et al., 2015). The gut microbiota produces signaling molecules like SCFAs by metabolizing polyphenols, influencing the synthesis of hippuric acid (HA). HA levels correlate with age-related phenotypes such as frailty and cognitive impairment (Ticinesi et al., 2023). Microbiota metabolites like hippuric acid (HA) may indirectly affect aging-related phenotypes through systemic pathways (e.g., inflammation or renal function) (Ticinesi et al., 2023). In individuals with metabolic disorders such as obesity and type 2 diabetes, the reduction in SCFA-producing microbiota directly leads to decreased levels of these beneficial metabolites. This not only compromises intestinal barrier function but also deprives muscle and neural tissues of crucial energy substrates and regulatory signals, exacerbating energy metabolism imbalance and disease progression (Kasubuchi et al., 2015). Research by An et al. (2021) further indicates that age influences microbial metabolic capacity. The concentration of SCFAs produced by the microbiota of elderly individuals after carbohydrate utilization is comparable to or even higher than that in adults, suggesting that their microbiota possesses distinct metabolic kinetics or compensatory mechanisms.

SCFAs, particularly butyrate, function not only as energy substrates and ligands for G protein-coupled receptors but also as potent endogenous inhibitors of histone deacetylases. Butyrate non-competitively inhibits class I and class IIa histone deacetylases, including HDAC1, HDAC2, HDAC3, and HDAC8, with half-maximal inhibitory concentrations ranging from 0.5 to 2 mM. Studies have shown that intragastric administration of butyrate at 200 mg/kg significantly reduces HDAC3 expression in inflamed tissues and increases the lysine acetylation levels of STAT1 and NF-κB p65. By inhibiting histone deacetylase activity, butyrate induces a relaxed chromatin state and increases the acetylation levels of histones H3 and H4, thereby regulating the transcription of genes involved in inflammation, immune modulation, mitochondrial function, and cellular senescence (Hu et al., 2024). This epigenetic regulatory mechanism holds significant implications for the pathophysiology of frailty. Histone deacetylase activity enhances the transcriptional activity of NF-κB p65 by deacetylating its lysine residues. Butyrate-mediated HDAC inhibition maintains the deacetylated state of NF-κB p65, reduces its transcriptional activity, and consequently suppresses the expression of pro-inflammatory cytokines such as TNF-α, IL-6, and IL-1β. A study by Peng et al. demonstrated that butyrate primarily blocks the NF-κB pathway by inhibiting HDAC8 and enhances Slc26a3 expression, thereby improving intestinal epithelial barrier function (Peng et al., 2024). In a bovine mammary epithelial cell model, butyrate treatment reduced HDAC3 expression, blocked NF-κB activation, and inhibited the production of inflammatory cytokines. In the context of frailty, short-chain fatty acid deficiency leads to increased histone deacetylase activity, resulting in excessive NF-κB activation and the persistence of chronic low-grade inflammation, also known as inflammaging (Wang et al., 2023).

Foxp3 is the master transcription factor of regulatory T cells, and its expression is finely regulated by histone acetylation levels. Short-chain fatty acids, through HDAC inhibition, increase histone H3 acetylation at the Foxp3 gene promoter and enhancer regions, promote Foxp3 transcription, and thereby maintain regulatory T cell differentiation and immunosuppressive function. Studies have confirmed that butyrate at 5 mM significantly increases the expression of Foxp3 and Ezh2, increasing the induction rate of CD4+Foxp3+ regulatory T cells by approximately 2 to 3 fold, with statistical significance (Furusawa et al., 2013). Chromatin immunoprecipitation sequencing analysis further revealed that butyrate treatment induces regulatory T cell polarization by enhancing histone H3 acetylation at the Foxp3 gene locus promoter and conserved non-coding sequence regions. Under short-chain fatty acid-deficient conditions, Foxp3 expression declines, regulatory T cell function is impaired, immune tolerance is disrupted, and inflammatory responses are exacerbated, accelerating frailty progression (Cao et al., 2018). Butyrate also regulates mitochondrial function through epigenetic mechanisms. Butyrate treatment increases histone H3 acetylation at the promoter region of PGC-1α, a master regulator of mitochondrial biogenesis, upregulates its expression, and subsequently promotes mitochondrial DNA replication and the transcription of oxidative phosphorylation-related genes. Studies have shown that butyrate at 200 mg/kg for 8 weeks significantly reduces plasma glucose levels in db/db diabetic mice, reverses the expression levels of PGC-1α and p-AMPK in the kidneys, and improves lipid accumulation and mitochondrial function. *In vitro* experiments further confirmed that butyrate intervention reverses high glucose-induced changes in ROS and ATP levels and increases the protein expression of PGC-1α and p-AMPK (Yu et al., 2023). Additionally, butyrate modulates the epigenetic status of mitochondrial fusion-fission related genes, including MFN1, MFN2, and DRP1, thereby maintaining mitochondrial network homeostasis. Short-chain fatty acid deficiency leads to impaired mitochondrial biogenesis and dynamic imbalance, representing an important mechanism of frailty-related energy metabolism disorders (Hu et al., 2020).

Short-chain fatty acids regulate the expression of cellular senescence-related genes through epigenetic mechanisms. Studies have shown that butyrate inhibits the transcription of senescence-associated genes such as p16INK4a and p21WAF1/Cip1, thereby delaying cellular senescence (Takeuchi et al., 2010). This effect is inversely correlated with the enrichment of butyrate-induced histone H3K9ac and H3K27ac at the promoter regions of senescence-associated genes. In the context of frailty, short-chain fatty acid deficiency may accelerate epigenetic aging and promote multisystem functional decline. The epigenetic regulatory mechanisms of short-chain fatty acids provide new targets for frailty intervention, including supplementation with fermentable fiber to increase endogenous short-chain fatty acid production, direct supplementation with butyrate or its analogues, and the development of specific histone deacetylase inhibitors (Berni Canani et al., 2012). These strategies may reverse short-chain fatty acid deficiency-related inflammation, immune dysfunction, and mitochondrial damage by restoring epigenetic homeostasis, thereby delaying or reversing the progression of frailty.

**Figure 2:**
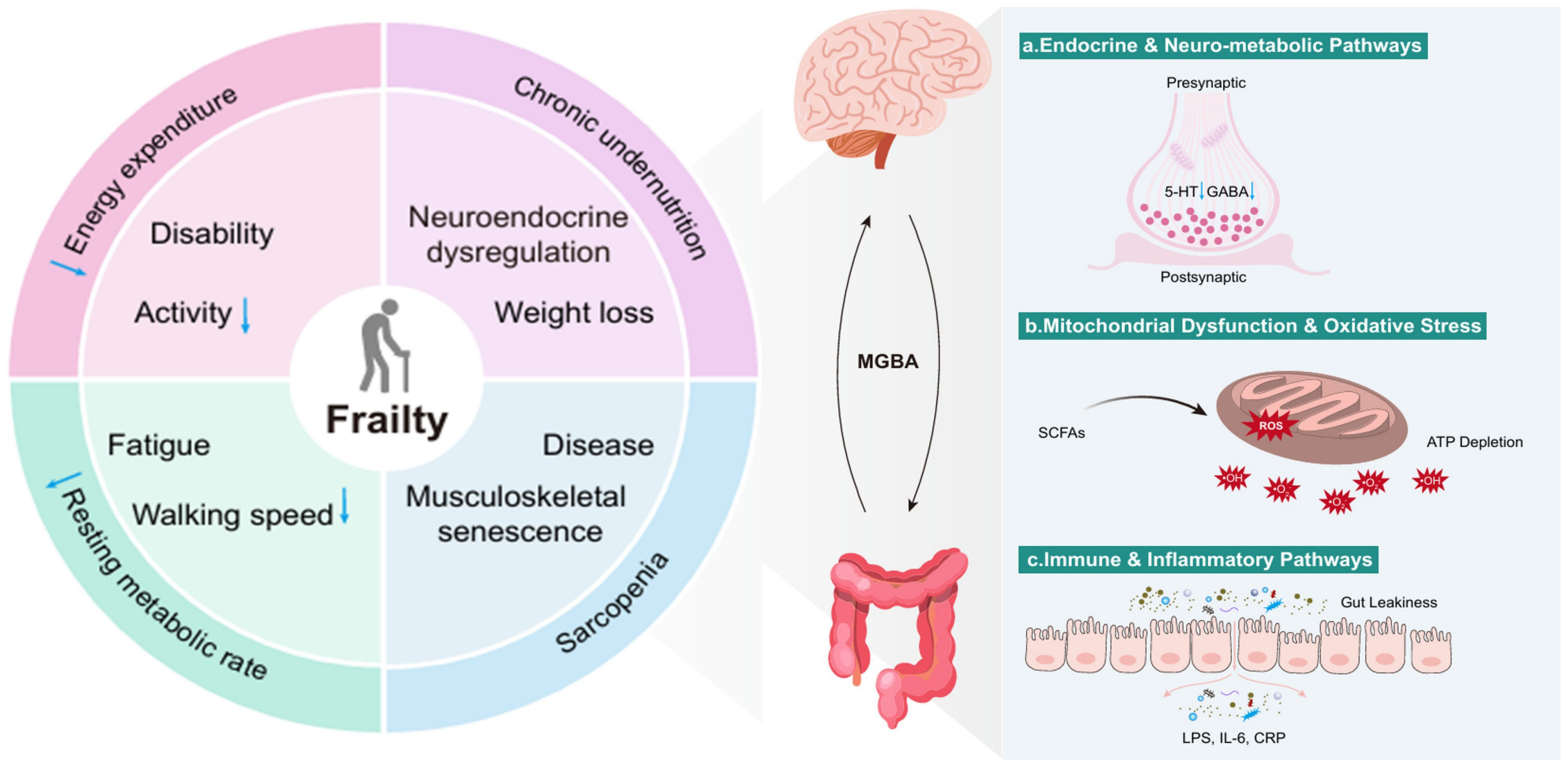
Mechanisms linking gut dysbiosis to frailty and cognitive decline in the elderly. Gut microbiota dysbiosis contributes to frailty and cognitive impairment through three major pathways: (1) metabolic and endocrine disturbances via microbial metabolites including SCFAs; (2) mitochondrial dysfunction and oxidative stress induced by microbial toxins; and (3) chronic low-grade inflammation triggered by abnormal immune activation. These pathways collectively drive multisystem dysfunction, muscle loss, and cognitive decline in frail older adults.

These findings collectively indicate that the gut microbiota profoundly engages in the physiological processes of frailty through endocrine and neurometabolic pathways. Targeting the regulation of the HPA axis, neurotransmitters, and SCFAs may offer novel perspectives for frailty prevention and treatment. Future research should further elucidate the specific mechanisms of these pathways to guide intervention strategies. In summary, MGBA mediates the pathogenesis of frailty through immune-inflammatory, mitochondrial dysfunction, and neurometabolic pathways, with its integrated interactive framework depicted in **Figure 2**. In contrast to the broader systemic mechanisms discussed above, endocrine and neurometabolic pathways constitute the core of MGBA specific communication. These pathways involve the direct stimulation of vagal nerve endings by microbial metabolites such as short-chain fatty acids, the regulation of hypothalamic-pituitary-adrenal axis activity by gut microbes, and neuroactive substances produced or modulated by gut microbiota including serotonin, gamma-aminobutyric acid, and kynurenine. These mechanisms do not depend on systemic inflammatory circulation. Instead, they form a direct and rapid regulatory network from the gut microbiota to the brain through neural, endocrine, and local metabolic signals, thereby playing a unique role in the development and progression of frailty.

In summary, gut dysbiosis promotes the development of frailty through two interrelated yet conceptually distinguishable pathways. The first pathway involves systemic, multi-organ aging biological effects, primarily including intestinal barrier dysfunction, systemic low-grade inflammation, systemic oxidative stress, and mitochondrial decline. These processes are triggered by gut dysbiosis and broadly affect multiple organ systems including muscle, liver, and brain. The second pathway comprises MGBA-specific neuroendocrine signaling networks, including vagus nerve activation, hypothalamic-pituitary-adrenal axis regulation, and direct actions of neuroactive metabolites such as short-chain fatty acids and serotonin. These mechanisms constitute a direct communication route between the gut microbiota and the central nervous system. In practice, these two pathways are highly overlapping. Systemic inflammation can exacerbate neuroinflammation, and metabolic disturbances can also affect neurological function. However, clarifying this conceptual distinction is crucial for guiding future research. Interventions targeting systemic pathways, such as broad-spectrum anti-inflammatory treatments, may be effective but lack specificity. In contrast, interventions targeting MGBA-specific pathways, such as vagus nerve stimulation or specific neuroactive probiotics, hold promise for more precise frailty management. The mechanisms reviewed in this article illustrate how these two pathways work synergistically to shape the multidimensional pathophysiological landscape of frailty.

## 4 Therapeutic intervention strategies targeting the gut microbiome

In recent years, intervention strategies targeting the gut microbiome have garnered significant attention in frailty management. These encompass multi-level approaches ranging from lifestyle modifications to cutting-edge technologies, aiming to improve frailty states by reshaping microbial ecosystems (Carrillo et al., 2022; Lim and Nam, 2023). Existing evidence primarily stems from randomized controlled trials, observational studies, and animal models, providing preliminary support for clinical practice. However, intervention efficacy varies due to strategy type, population heterogeneity, and complex mechanisms. The following sections systematically evaluate the strength, consistency, and limitations of evidence for each strategy, with detailed study information presented in **Table 2**.

**Table 2:** Summary of gut microbiota-targeted intervention strategies.

Author/year	Subjects	Intervention type	Sample characteristics	Intervention details	Main results	Ref.
Ghosh et al., 2020	Frail older adults	Dietary intervention (Mediterranean diet)	612 European adults aged 65–79 years	1-year duration	Increased abundance of beneficial bacteria such as Faecalibacterium prausnitzii, reduced inflammatory markers like C-reactive protein (CRP), and improved cognitive function	(Ghosh et al., 2020)
Zhang et al., 2025	General adult population	Cross-sectional study (observational)	6283 adults	–	High dietary quality associated with lower frailty index and synergy with oral microbiome diversity	(Zhang et al., 2025)
Li et al., 2022	Older adults	Observational analysis	15179 older adults	–	Higher dietary live microbe intake associated with 22.1% reduction in frailty prevalence (OR = 0.779)	(Li et al., 2025)
Allen et al., 2018	Healthy adults aged 20-45	Exercise	–	–	Increased SCFA concentrations post-exercise	(Allen et al., 2018)
Mei et al., 2021	Healthy young volunteers (aged 20-35)	Probiotics (Lactobacillus casei LTL1879)	20 volunteers	3-week supplementation	Increased total antioxidant capacity by 52.70% (p<0.0001), reduced inflammatory factors like TNF-α by 32.38% (p<0.05), promoted proliferation of beneficial bacteria like Lactobacillus	(Mei et al., 2021)
Castro-Herrera et al., 2021	Elderly nursing home residents (mean age 86 years)	Probiotics (Lacticaseibacillus rhamnosus GG and Bifidobacterium animalis subsp. lactis BB-12)	60 residents	Daily dose of 1.3-1.6 x 10^9 CFU, up to 12 months	No significant effect on most immune markers, but improved influenza vaccine response (seroconversion rate 47% in probiotic group vs. 15% in placebo, p=0.04)	(Castro-Herrera et al., 2021)
Theou et al., 2019	Nursing home elderly residents (mean age 75.3 years)	Prebiotics (containing inulin and fructooligosaccharides)	50 residents	13-week daily supplementation	Significant reduction in frailty index (from 0.22 to 0.20, p<0.001), while placebo group increased (from 0.23 to 0.24, p=0.012), with an average reduction of 0.02 (equivalent to 1.1 deficits)	(Theou et al., 2019)
Buigues et al., 2016	Older adults aged ≥65 in nursing homes	Prebiotics (Darmocare Pre® containing inulin and oligofructose)	60 residents	13-week daily supplementation	Significant improvement in grip strength (from baseline 10.6 ± 8.2 kg to 12.4 ± 3.2 kg, p<0.05), reduced fatigue scores (p<0.01)	(Buigues et al., 2016)
Yang et al., 2024	Community-dwelling older adults	Prebiotics (mixture of inulin and oligofructose)	200 older adults	13-week supplementation	Improved frailty status (p<0.05), increased grip strength, walking speed, and body fat percentage, modulated renal function indicators	(Yang et al., 2024)
Wang et al., 2022	D-galactose-induced aging mouse model	Nutritional intervention (2’-Fucosyllactose)	Mouse model	8-week intervention	Improved oxidative stress (e.g., reduced serum MDA) and intestinal barrier function	(Wang et al., 2022)
Dong et al., 2024	Frail patient microbiota transplanted into germ-free mice	Probiotics (Lactobacillus plantarum BFS1243)	Germ-free mice	Supplementation with Lactobacillus plantarum BFS1243	Reversed dysbiosis, enhanced intestinal barrier integrity (e.g., increased ZO-1 expression), prolonged forced swimming time (p<0.05)	(Dong et al., 2024)
Tran et al., 2019	Frail older adults	Prebiotic mixture (containing wheat dextrin, resistant starch, etc.)	–	26-week supplementation	Increased abundance of beneficial bacteria like Ruminococcaceae and Parabacteroides, reduced inflammatory factor CXCL11 levels (p<0.05)	(Tran et al., 2019)
Montalto et al., 2023	Very old frail patients (median age 88 years)	Fecal Microbiota Transplantation (FMT)	43 patients	FMT for recurrent Clostridioides difficile infection	Success rates of 77% (first infusion) and 88% (overall), frailty assessment associated with treatment failure risk (p<0.01)	(Montalto et al., 2023)
Jørgensen et al., 2020	Severely frail older patients	Fecal Microbiota Transplantation (FMT)	4 patients	Via nasojejunal tube or capsules, some required repeated infusions	Clinical improvement, highlighting safety in frail populations	(Jørgensen et al., 2020)
Zhu et al., 2024	Young and old mice (n=6 per group)	Fecal Microbiota Transplantation (FMT)	Mice	Bidirectional FMT	Improved frailty phenotype in old mice (e.g., increased grip strength p=0.036, prolonged running time), restored intestinal barrier, reduced systemic inflammation (serum TNF-α decreased p=0.002)	(Zhu et al., 2024)

Dietary interventions, particularly the Mediterranean diet pattern, have been demonstrated to effectively modulate the gut microbiome and alleviate frailty. Multiple studies consistently indicate that high dietary quality is independently associated with reduced frailty indices. For instance, the NU-AGE trial by Ghosh et al. (2020) demonstrated that a one-year Mediterranean diet intervention increased the abundance of beneficial bacteria (e.g., Faecalibacterium prausnitzii) and was associated with reduced inflammatory markers (e.g., CRP) and improved cognition. Similarly, cross-sectional analyses by Zhang et al. (2025) and Li et al. (2025) further supported the association between dietary quality and reduced frailty prevalence, with synergistic effects observed between diet and microbial diversity. Collectively, these findings indicate that dietary modifications serve as an effective strategy for frailty management by promoting beneficial microbiota and suppressing inflammatory pathways.

Physical exercise, as another non-pharmacological intervention, can influence the progression of frailty by modulating the gut microbiome. In human studies, aerobic exercise such as 6 weeks of moderate-intensity training increases the abundance of SCFA producers like Faecalibacterium and improves microbiome function (Shin et al., 2019). Research by Allen et al. (2018) demonstrates that aerobic exercise elevates SCFA concentrations, while animal studies suggest exercise-induced microbial changes (e.g., increased Bifidobacterium) correlate with reduced inflammation and preserved muscle function.

Probiotics, prebiotics, and synbiotics demonstrate significant potential in frailty interventions by directly modulating the gut microbiome. Regarding probiotic interventions, Mei et al. (2021) reported that short-term supplementation with Lactobacillus casei enhances antioxidant capacity and reduces inflammatory markers. Conversely, Castro-Herrera et al. (2021) observed that specific strains (e.g., Lacticaseibacillus rhamnosus GG) in elderly populations, while having no effect on most immune markers, enhanced vaccine responses, suggesting differential immunomodulatory pathways. In contrast, evidence for prebiotics is more consistent: Studies by Theou et al. (2019), Buigues et al. (2016), and Yang et al. (2024) all demonstrated that prebiotic supplementation significantly reduced frailty indices, improved grip strength, and alleviated fatigue, with substantial effect sizes. Studies by Dong et al. (2024) and Tran et al. (2019) indicate that prebiotic/probiotic interventions can reverse dysbiosis, strengthen the intestinal barrier, and reduce inflammation. Overall, probiotic/prebiotic strategies alleviate frailty through SCFA metabolism and immune modulation, but strain selection, dosage, and individual variability remain key challenges for optimizing interventions.

Fecal microbiota transplantation (FMT) and other cutting-edge strategies offer innovative approaches to frailty management by directly reshaping the gut microbiota. Clinical studies demonstrate the feasibility of FMT in frail populations: Montalto et al. (2023) and Jørgensen et al. (2020) reported that FMT improves clinical outcomes, with frailty severity predicting treatment response; Animal studies, such as that by Zhu et al. (2024) demonstrated through bidirectional FMT that transplanting young microbiota reverses frailty phenotypes in aged mice (e.g., improved grip strength and reduced inflammation), involving mechanisms of increased SCFAs and inhibition of the TLR4/NF-κB pathway. However, these studies have small sample sizes and primarily focus on infected patients; the efficacy and safety of FMT for primary frailty require validation through large-scale RCTs. Notably, while both probiotics/prebiotics and FMT target the microbiome, the latter directly reshapes the entire microbiota and may be more suitable for severely frail individuals, whereas the former is more readily implemented in the prevention phase.

In summary, intervention strategies targeting the gut microbiota present a multi-tiered framework: probiotic/prebiotic interventions have the most robust evidence, dietary and exercise strategies require further mechanistic research, while FMT holds significant potential but carries higher risks. The common core of these strategies lies in alleviating inflammation and dysfunction through microbe-host interactions. Future research must address individual variations (e.g., genetics and comorbidities) and clarify causal sequencing through longitudinal designs to advance precision aging management.

## 5 Challenges and outlook

Current research on the role of the MGBA in the pathogenesis of frailty still faces significant limitations. These challenges primarily stem from the heterogeneity of study designs: most evidence derives from cross-sectional studies, making it difficult to infer causality; small sample sizes and substantial population variability (e.g., age, comorbidities, and medication use) lead to inconsistent results; Lack of standardized microbiome analysis protocols impedes the clinical translation of biomarkers; Intervention strategies (e.g., probiotics or dietary modifications) exhibit variable efficacy due to individual differences (e.g., genetic background and underlying diseases), and residual confounding factors cannot be fully eliminated. These limitations collectively underscore the imperative to advance research from associative studies toward causal mechanisms.

Although the role of the microbiota-gut-brain axis in the mechanisms of frailty has garnered attention, its clinical translation still faces significant gaps. In terms of evidence, most probiotic and prebiotic intervention studies have small sample sizes, short follow-up durations, and wide confidence intervals in effect estimates. Although the Mediterranean diet has been shown to improve frailty indices, the effect size is modest and its generalizability remains to be validated. Regarding biomarkers, machine learning models used to distinguish between frail and non-frail individuals are often based on single-center, small-sample data lacking external validation. Moreover, high testing costs, non-standardized procedures, and the complexity of data analysis further limit their clinical applicability. With respect to intervention strategies, although butyrate has shown potential for preventing muscle atrophy in animal studies, its low oral bioavailability, rapid metabolism, undetermined optimal dosage, and unclear safety profile preclude its routine use in frailty prevention and treatment at present.

Current research findings do exhibit a certain degree of heterogeneity, which primarily stems from differences in study designs and assessment tools. For instance, the Fried frailty phenotype focuses on physical functional deficits, whereas the frailty index is based on the accumulation of health deficits, and the different metrics of these two tools may influence the analysis of microbial association characteristics. Furthermore, significant differences exist between 16S rRNA sequencing and metagenomic sequencing in terms of taxonomic resolution and functional annotation capacity, which may affect the sensitivity of detecting gut microbiota differences. Insufficient sample sizes, as well as variations in confounding factors such as geographic location, dietary patterns, and medication use, may also interfere with the characterization of gut microbiota baselines. Regarding population characteristics, differences in the cut-off values used to define frailty may lead to substantial distinctions in the physiological reserves of the included populations, while variations in age range, sex distribution, comorbidity profiles, and ethnicity may also influence the baseline composition of gut microbiota. Some studies have suggested that the association between microbial species and metabolites with frailty is more pronounced in females, indicating that sex may be an important stratifying factor. To advance clinical translation, future studies are encouraged to consider the following stratification strategies, conducting sex-stratified analyses and reporting the associations between gut microbiome and frailty index separately for males and females; shifting the focus from taxonomic classification toward metabolic pathway stratification and attempting to establish a frailty-associated metabolic feature score to reduce bias arising from taxonomic heterogeneity; and using Mendelian randomization methods to identify specific bacterial genera with causal relationships and constructing polygenic risk scores to identify high-risk individuals. Of note, composite scores based on microbial features have already demonstrated independent predictive value for mortality, suggesting that such stratification tools hold potential for identifying high-risk populations.

Future research should focus on filling existing gaps to advance the precision application of MGBA-targeted interventions. Future efforts should include large-scale longitudinal multi-omics cohorts integrating genomic, metabolomic, and microbiome data to elucidate the temporal causal mechanisms of MGBA pathways (e.g., roles of SCFAs or inflammatory pathways) (Chatterjee et al., 2025); prospective designs and randomized controlled trials (RCTs) are essential for validating dietary indices (e.g., DI-GM) or probiotic intervention efficacy (Gan et al., 2025; Wei et al., 2025); Additionally, personalized strategies (e.g., based on microbial profiling) and interdisciplinary integration (e.g., artificial intelligence with wearable devices) can optimize intervention outcomes (Chatterjee et al., 2025; Gan et al., 2025; Wei et al., 2025). By addressing these directions, MGBA research holds promise for delivering translational biomarkers and intervention strategies for frailty prevention and management.

## 6 Conclusion

This review systematically elucidates the central role of the MGBA in the pathogenesis of frailty, emphasizing its function as a critical bridge linking gut dysbiosis to multisystem physiological decline. By integrating clinical evidence and mechanistic studies, we reveal that the MGBA not only drives frailty progression through immune-inflammatory, mitochondrial metabolic, and neuroendocrine pathways but also demonstrates significant potential as novel biomarkers and precision therapy targets. Multidimensional interventions targeting the MGBA—such as dietary modifications, probiotic supplementation, and lifestyle optimization—effectively disrupt the vicious cycle between frailty and dysbiosis, offering innovative strategies for frailty prevention and management while advancing healthy aging. This review contributes by establishing a comprehensive framework for frailty within the MGBA perspective, laying a theoretical foundation for future research and clinical practice. This review systematically elucidates the central role of the MGBA in the pathogenesis of frailty, emphasizing its function as a critical bridge linking gut dysbiosis to multisystem physiological decline. By integrating clinical evidence and mechanistic studies, we reveal that the MGBA not only drives frailty progression through immune-inflammatory, mitochondrial metabolic, and neuroendocrine pathways but also demonstrates significant potential as novel biomarkers and precision therapy targets. Multidimensional interventions targeting the MGBA—such as dietary modifications, probiotic supplementation, and lifestyle optimization—effectively disrupt the vicious cycle between frailty and dysbiosis, offering innovative strategies for frailty prevention and management while advancing healthy aging.

This review contributes by establishing a comprehensive framework for frailty within the MGBA perspective, laying a theoretical foundation for future research and clinical practice. It should be noted that the current evidence is derived primarily from cross-sectional studies and animal models, and the establishment of causal relationships as well as clinical translation still face significant challenges. Randomized controlled trials of probiotic or prebiotic interventions generally have small sample sizes and short follow-up durations, while lifestyle interventions such as the Mediterranean diet show only modest effects on frailty indices with inconsistent findings across studies. Furthermore, no randomized controlled trial of fecal microbiota transplantation for primary frailty has been published to date. In addition, the substantial heterogeneity in microbiome research and inadequate control of confounding factors limit the generalizability of the conclusions. Therefore, interventions targeting the microbiome-gut-brain axis should currently be considered as adjunctive rather than replacing approaches to conventional frailty management, which includes exercise, nutritional assessment, and polypharmacy adjustment. The epigenetic regulatory mechanisms of butyrate supplementation represent a novel research direction for frailty intervention, but this strategy remains at the preclinical stage, and its efficacy and safety require validation through large-scale randomized controlled trials. Future research should integrate longitudinal multi-omics data, establish standardized analytical workflows, and promote the clinical translation of microbiome-based biomarkers.
